# High-efficiency terahertz devices based on cross-polarization converter

**DOI:** 10.1038/s41598-017-18013-6

**Published:** 2017-12-20

**Authors:** Huan Zhao, Xinke Wang, Jingwen He, Jinying Guo, Jiasheng Ye, Qiang Kan, Yan Zhang

**Affiliations:** 1 Department of Physics, Capital Normal University, Beijing Key Laboratory of Metamaterials and Devices, Key Laboratory of Terahertz Optoelectronics, Ministry of Education, and Beijing Advanced Innovation Center for Imaging Technology, Beijing, 100048 P. R. China; 2 Beijing Institute of Satellite Information Engineering and State Key Laboratory of Space-Ground Integrated Information Technology and State Key Laboratory of Space-Ground Integrated Information Technology, Beijing, 100086 P. R. China; 3 Key Laboratory of Semiconductor Materials Science, Institute of Semiconductors, Chinese Academy of Sciences, Beijing, 100083 P. R. China

## Abstract

Metasurface-based devices have been investigated intensively because of their attractive properties but these devices generally suffer from low efficiency. Here we demonstrate several high-efficiency terahertz (THz) devices based on cross-polarization converters that is composed of bilayer metasurface-based structures. The converter can transfer the polarization states of transmitted THz waves from the *x*-direction into the *y*-direction with an experimental conversion efficiency of 85%. This high-efficiency transfer mechanism is investigated in detail. Furthermore, this kind of devices can be fabricated easily. A THz metalens is designed and fabricated and its focusing and imaging properties are investigated experimentally. A pure phase THz hologram that can generate different images on different propagation planes is also designed and the image reconstruction capabilities of the phase holograms are demonstrated experimentally. The performance levels of all designed devices show excellent agreement between the theoretical expectations and the corresponding experimental results. This technology may pave the way towards practical applications of such metasurface devices.

## Introduction

Polarization is a basic characteristic of electromagnetic waves and conveys valuable information during signal transmission. Cross-polarization converters (CPCs) can be used to rotate a linear polarization state into an orthogonal state. The conventional methods that are used to change the polarization states of electromagnetic waves are generally based on material properties such as birefringence or total internal reflection effects^1^. However, the limited permittivity and permeability ranges of natural materials impose significant limitations in terms of working wavelength and efficiency. The metasurface, which is a type of artificial planar structure composed of arrays of sub-wavelength antennas, has been gaining increasing research attention because of properties that include its small size, light weight, and low cost. Perhaps the most interesting property of this type of structure is that introduction of an abrupt interfacial phase discontinuity allows a metasurface to modulate both the phase and the amplitude of electromagnetic waves from the visible range to microwave frequencies^2–17^. Metasurface-based polarization control devices, such as polarization converters^18–22^, half-wave plates^23,24^, and quarter-wave plates^25,26^, have been investigated intensively in recent years, and the lack of suitable natural materials for terahertz (THz) radiation polarization conversion means that metasurface-based polarization manipulation devices are highly desirable. In particular, because of the complex amplitude and phase modulation functionalities of cross-polarization, the linear, reciprocal, and passive metasurface with its infinitesimal thickness has also been used to fabricate various devices for wavefront modulation applications. However, the single-layer metasurface structure, which largely uses localized surface plasmonic resonances^27–30^, generally suffers from either limited bandwidth or high losses. It has previously been proven that the efficiency of a CPC composed of a single-layer metasurface is limited to a maximum of 50%^27^.

Considerable efforts have been made to improve CPC efficiency. One approach involved use of a dielectric medium rather than a metal^31,32^. Lin *et al*. used their versatile patterning technique to create a suite of planar optical elements. By patterning a 100-nm-thick silicon layer into a dense arrangement of nano-antennas, they fabricated gratings, lenses, and axicons, which can be used to add shapes to propagating light beams^31^. The efficiency of these devices reached 75% at 500 nm. Khorasaninejad *et al*. showed that arrays of nanoscale TiO fins can function as high-end optical lenses, with efficiencies as high as 86%, 73%, and 66% at working wavelengths of 405, 532, and 660 nm, respectively^32^. However, the complex processing method required precludes any realistic application of these types of devices. Additionally, only light with circular polarization can be modulated using these devices. A single layer metasurface for linearly polarized light has been demonstrated in the middle infrared for phase and polarization control with high transmission efficiency^33^. However, this technology cannot be easily scaled to the THz region in which a 300 μm high dielectric microstructure should be fabricated, which is still a big challenge.

Another approach to improve CPC efficiency is the use of a multilayer structure based on the Fabry-Pérot (F-P) resonance. A regular bilayer structure containing a metasurface layer, a gold ground reflecting layer and a sandwiched dielectric layer was used to form a broadband reflection mode CPC. A bilayer structure based on L-shaped antennas was used to produce almost complete conversion of the linear polarization of reflected light in the infrared range, with a mean polarization conversion efficiency of approximately 95%^34^. An ultra-wideband CPC consisting of two pairs of L-shaped metallic patches that were printed on a substrate backed by a metallic ground layer was also proposed. The polarization conversion efficiency of this CPC was more than 90% in the 7.8–34.7 GHz frequency range^35^. However, the reflective devices used involve the use of complex optical setups that may be inconvenient for practical applications. Recently, transmission mode CPCs have been attracting increasing interest. Using a three-layer structure composed of two orthogonal gold grating layers and a central gold antenna layer, a broadband and efficient CPC for transmitted THz waves has been demonstrated and anomalous refraction was also realized using this type of device^18^. An efficient three-layer structure has been shown to act as an efficient transmission mode CPC in the THz band. Using three layers of C-shaped gold antennas with 45° relative rotation between the layers, a transfer efficiency of 76% was achieved and a device was also designed to generate nondiffracting Bessel beams^21^. However, the fabrication of these three-layer structures is quite complex, involving multiple spin-coating, photolithography, and metallization processes, which made the development of multilayer metasurface-based CPCs very challenging. Bilayer metasurface CPCs require rather simpler fabrication processes. A bilayer transmission-mode CPC has been realized for operation in the visible light range using an array of V-shaped gold antennas and Babinet-inverted apertures on a gold film; however, the polarization efficiency of this CPC is less than 40%^19^.

In this work, an easily fabricated high-efficiency bilayer CPC metasurface structure has been demonstrated experimentally. This structure consists only two layers, a subwavelength grating and a subwavelength antenna array. The relative position of two layers is not so strict. However, it can convert the polarization state of a transmitted THz wave from the *x*-direction into the *y*-direction with experimental conversion efficiency of 85%. The mechanism that produces the high efficiency of the structure is investigated in detail. A metalens based on this structure is then designed and fabricated, and its focusing and imaging properties are investigated experimentally. A pure phase hologram that can generate different images on different planes is also designed and fabricated, and the image reconstruction capability of the phase hologram is demonstrated experimentally. The performances of all designed THz devices show excellent agreement between the theoretical predictions and corresponding experimental results, which indicates that the proposed two-layer metasurface structure can be used for high-efficiency THz device design. This structure will lead to improved approaches for the miniaturization and integration of THz systems.

## Design

**Figure 1:**
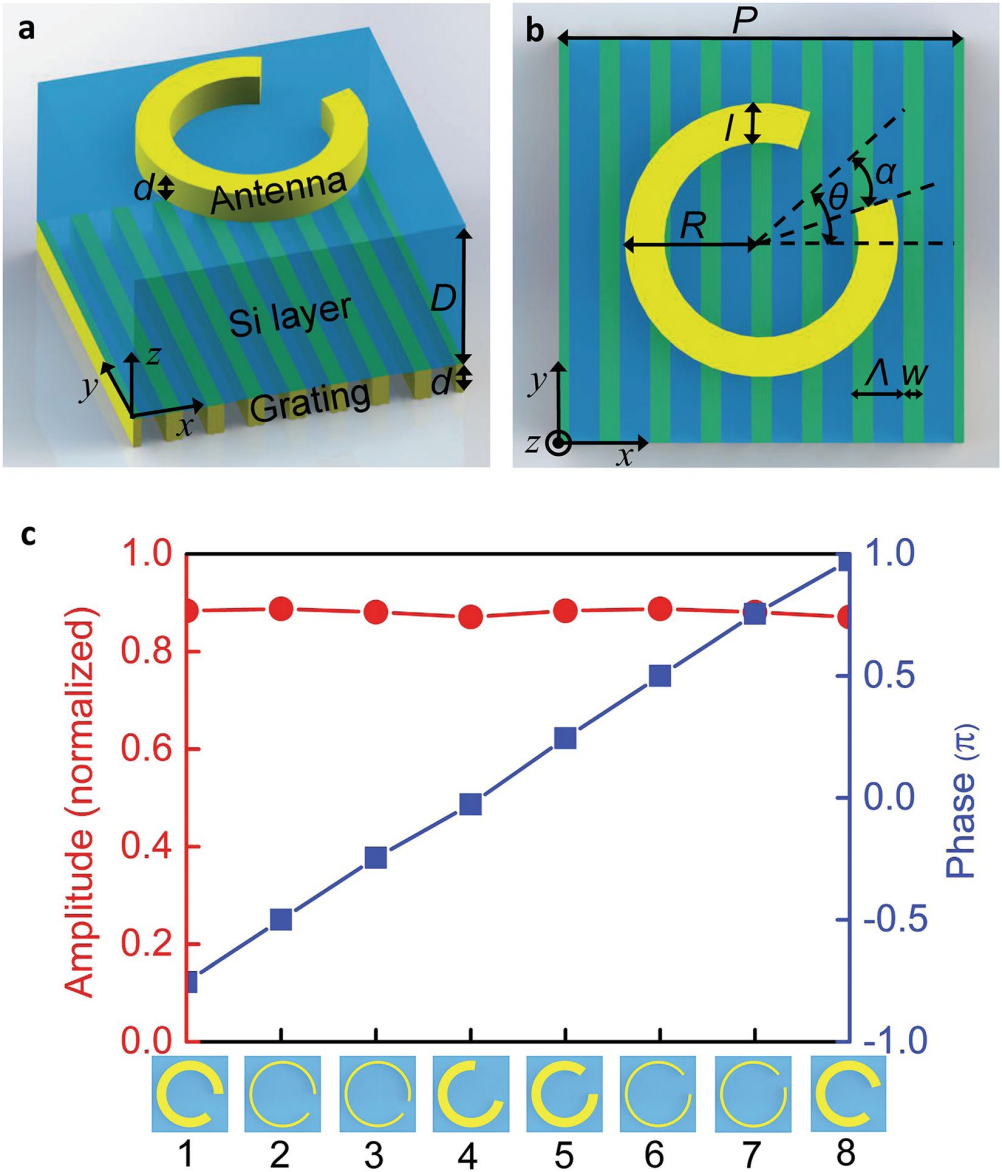
(**a**) Schematic view of the CPC. (**b**) Top view of the CPC. (**c**) Simulated scattering amplitudes and phases of the cross-polarized radiation from the individual CPCs. The amplitude is normalized to the incident *x*-polarized light. The silicon layer thickness *D* is 80 μm, while the thicknesses of both the grating layer and the antenna layer are represented by *d* with a value of 40 nm. The spacing between two neighboring CPC units is also 80 μm. The eight CPC units are composed of selected C-shaped antennas and identical gratings. The period *Λ* and width *W* of the grating are 10 μm and 4 μm, respectively. The first four antennas have opening angles of 2*α* = 50°, 37°, 15°, and 95°, with corresponding widths of *l* = 10, 3, 3, and 10 μm and angles of *θ* = −25°, −18.5°, −17.5°, and 37.5°, respectively. The other four antennas are mirror images of the first four antennas.

The basic structure proposed here consists of two physically separated metasurface layers. The top layer is an array of C-shaped gold antennas and the bottom layer is composed of gold gratings, with a silicon substrate at the center of the structure, as shown in Fig. 1(a). Both the antennas and the gratings have the same thickness *d* of 40 nm, and the silicon layer thickness *D* is 80 μm. Figure 1(b) shows the top view of the structure. The spacing P between two adjacent structures is also 80 μm. The width *W* and period *Λ* of the grating are 4 μm and 10 μm, respectively. Due to the period of the gold grating is much less than the wavelength of the incident light, the gold grating acts as a polarizer that can transmit the *x*-polarized radiation and reflect the *y*-polarized THz radiation almost completely. Therefore, the relative position between two layers is not so strict. The outer radius of the C-shaped antenna is R = 35 μm and the antenna width is either 10 μm or 3 μm. The symmetric axis of the C-shaped antenna is at an angle θ with respect to the *x*-axis. The opening angle of the antenna is 2*α*. When a linearly-polarized THz wave impinges on an antenna unit, both “symmetric” and “antisymmetric” modes can be excited, and a cross-polarized field is thus scattered by these two modes; the amplitude and phase of the cross-polarized field can be then determined using the geometrical parameters of the antennas^4,17,36^. By optimizing both the width and the opening angle of the C-shaped gold antenna, the phase of the transmitted *y*-polarized light can be modulated in the range between 0 and 2π with a constant phase difference of π/4, while the amplitude transmission is maintained at 0.9 at an operating frequency of 0.75 THz, as shown in Fig. 1(c). Commercial software FDTD Solutions based on finite-difference time-domain method is used to determine the optimal values of both the width and the opening angle. In the simulations, the metal is set to be a perfect electrical conductor, which is a reasonable assumption in the THz frequency range. The eight different C-shaped antennas that were selected are shown at the bottom of Fig. 1(c), and the parameters corresponding to these antennas are given in the caption for Fig. 1.

**Figure 2:**
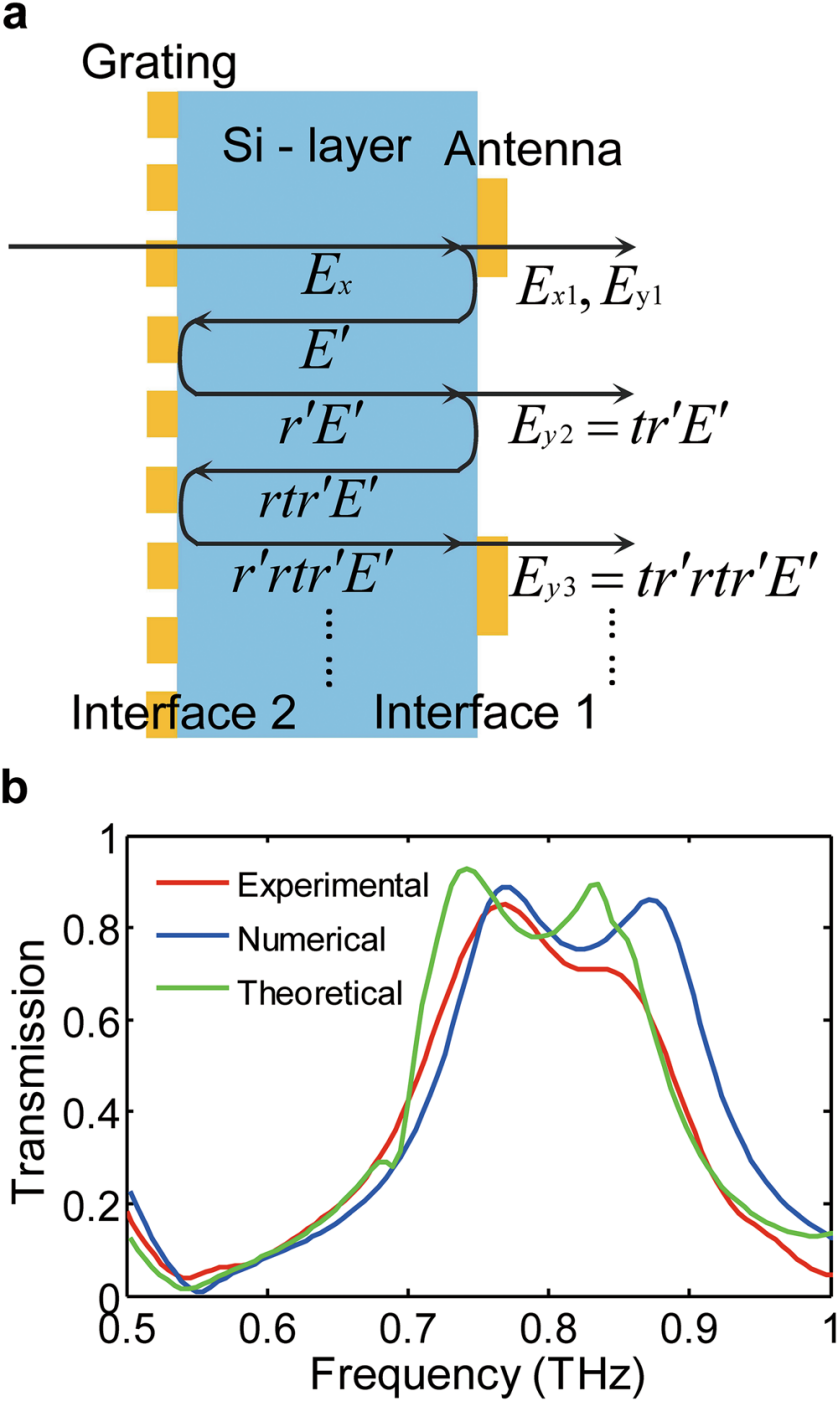
(**a**) Schematic of the components of the scattering paths in the CPC. The interface between the silicon layer and the antenna layer is designated Interface 1 and the interface between the silicon layer and the grating layer is Interface 2. *t* and *r* denote the amplitude transmission and reflection of the *y*-polarized wave at Interface 1, while *r*′ denotes the amplitude transmission of the *y*-polarized wave at Interface 2. (**b**) Theoretical, numerical and experimental results for the transmitted *y*-polarized radiation. The maximum transmission obtained in the experiments was 0.85 at 0.75 THz.

To clarify the mechanism behind the high-efficiency performance of the designed structure, a solution based on the F-P resonance mode is proposed. Figure 2(a) shows a schematic of the various parts of the scattering paths in the structure. The interface between the silicon layer and the antenna layer is designated Interface 1 and the interface between the silicon layer and the grating layer is designated Interface 2. Parameters *t* and *r* denote the amplitude transmission and reflection of the *y*-polarized wave at Interface 1, respectively, while r′ denotes the amplitude transmission of the *y*-polarized wave at Interface 2. Because the gratings are arranged along the *y*-direction, the y-polarized wave is reflected almost completely, so r′ is equal to 1. *E*_*x*_ denotes the incident *x*-polarized wave. When passing through the structure, the transmitted wave contains both *x*-polarized component *E*_*x1*_ and y-polarized component *E*_*y1*_, *E*′ represents the waves reflected by Interface 1. Because the *x*-polarized component of *E*′ is almost entirely transmitted by Interface 2, the x-polarized wave that is reflected by Interface 2 can be ignored; in contrast, the y-polarized component of *E*′ is almost entirely reflected by Interface 2 to generate *E*_*y*_′, which is then transmitted through Interface 1 as *E*_*y2*_. The output y-polarized wave *E*_*outy*_ is formed by the superposition of each *E*_*yi*,_ and the structure preformed like a F-P resonator, when the optical length of the resonator meets the resonant condition which is *2nd*/*λ* + *Δ* *φ*_*1*_ + *Δ* *φ*_*2*_ = *2m* *π*, where *λ* is the wavelength of incident light, *n* and *d* are reflective index and thickness of the Si layer, *Δ* *φ*_*1*_ and *Δ* *φ*_*2*_ are the phase modulation at Interface 1 and Interface 2 respectively, m is an integer. The constructive interference was achieved and the output *y*-polarized wave *E*_*outy*_ can be described as:

1 $${E}_{outy}=\sum _{i}{E}_{yi}={E}_{y1}+{E}_{y2}+{E}_{y3}+\cdots ={E}_{y1}+tr\text{'}{E}_{y\text{'}}[1+rtr\text{'}+{(rtr\text{'})}^{2}+{(rtr\text{'})}^{3}+\cdots ],$$

Eq. (1) can be simplified to give:

2 $${E}_{outy}=\sum _{i}{E}_{yi}={E}_{y1}+\frac{t}{1-r}{E}_{y\text{'}}.$$

Using Eq. (2), we can calculate the amplitude transmission of the *y*-polarized wave and compare it with both the numerical results and the experimental results, as shown in Fig. 2(b). We defined the amplitude polarization conversion efficiency as: *E*_*outy*_ */E*_*x*_. The values of *E*_*y1*_, *E*_*y*_′, *t* and *r* were determined via appropriate simulations. The figure shows that both the theoretical and numerical amplitude transmissions of the y-polarized waves are approximately equal to 0.88 at 0.75 THz. The designed CPC is fabricated on the two sides of an 80-μm-thick double-side polished silicon substrate using conventional photolithography and metallization processes. Because there are no strict requirements for the relative positioning of the gratings and the antennas, the fabrication procedure is relatively simple. The performance of the resulting CPC unit is characterized using a THz time domain spectroscopy system (THz-TDS). The maximum amplitude transmission is 0.85 at 0.75 THz, which indicates that the designed CPC performs with high efficiency. The three curves in the figure have similar shapes, thus indicating that the proposed model explains the high-efficiency mechanism well.

## Results and Discussion

**Figure 3:**
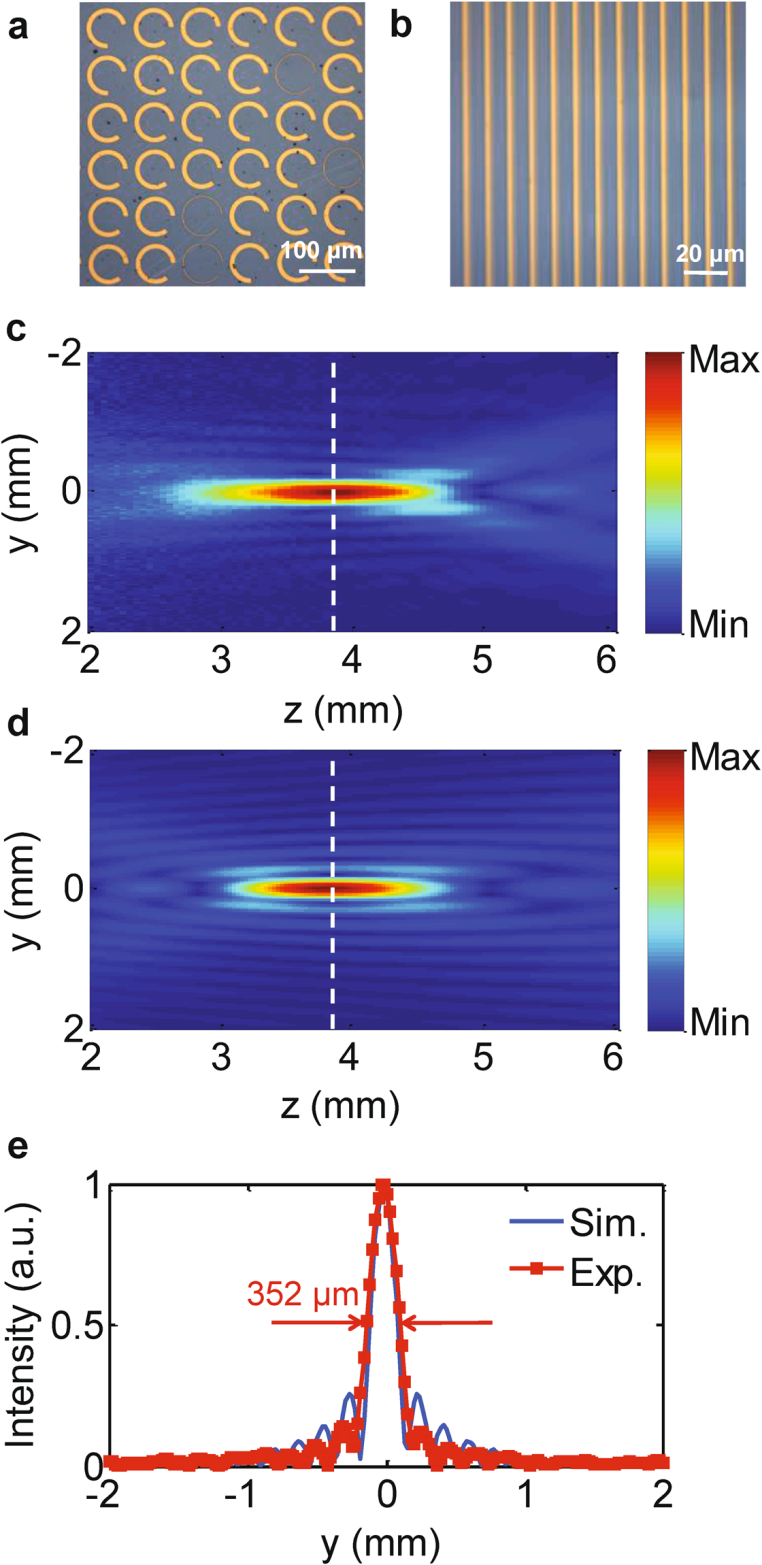
(**a**) Photograph of the C-shaped antenna side of the fabricated metalens, where the scale bar denotes 100 μm. (**b**) Photograph of the grating side of the fabricated metalens, where the scale bar denotes 20 μm. (**c**) Experimentally measured intensity distribution of the *y*-polarized light for the fabricated metalens on the *y*-*z* plane. The step length in the optical propagation direction was 0.5 mm. (**d**) Intensity distribution of the *y*-polarized light for the designed metalens along the *z*-direction, which was simulated using commercial software. (**e**) Intensity distributions along the white dashed lines shown in (**c**) and (**d**). The solid blue and dotted red curves represent the simulated and experimental results, respectively. The full width at half maximum in the experimental results is 352 μm.

**Figure 4:**
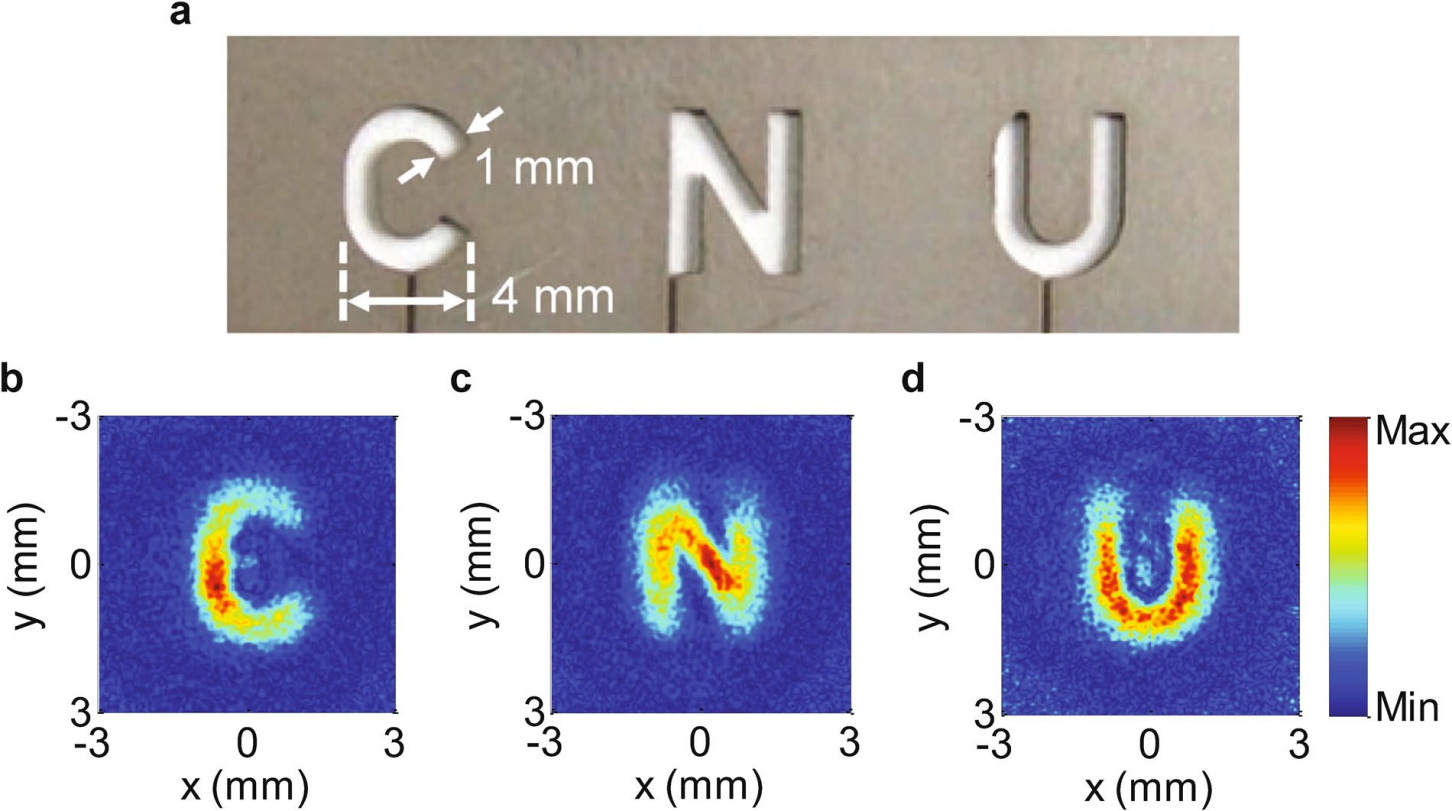
(**a**) Photograph of the object to be imaged. The size of each letter is 4 × 5 mm^2^ and the slit width is 1 mm. The letters were drilled on a stainless steel slice with a thickness of 0.3 mm. (**b**–**d**) Images of the three letters. The images are reduced based on the object-image relationship.

We have designed two high-efficiency THz devices based on the CPC metasurface structure: a metalens and a phase hologram device. For the metalens design, the phase distributions required for the lens at different positions can be readily obtained based on the equal optical path principle, as follows:

3 $$\phi (x,y)=\frac{2\pi }{\lambda }(f-\sqrt{{x}^{2}+{y}^{2}+{z}^{2}}).$$

where λ is the incident wavelength, which is set at 0.75 THz, and *f* is the focal length of the lens, which is 3.84 mm in this design. The phase value is wrapped within the range from 0 to 2π and is quantized into eight values, as shown in Fig. 1(c). A set of CPC structures is then selected based on the calculated phase distribution. The designed metalens has 128 × 128 cells, which corresponds to a geometrical area of 1.024 × 1.024 cm^2^, and the numerical aperture is 0.8, which is the same as the numerical aperture value given in ref.^32^. Figures 3(a) and 3(b) show the sections of the metalens on the top side and the bottom side of the substrate, respectively. The figure shows that both the C-shaped gold antennas and the grating have been fabricated well, with processing errors of less than 1 μm. A THz holographic imaging system is used to measure the field information for the *y*-polarized scattered THz wave. The intensity distribution of the *y*-polarized light on the *x-z* plane is as shown in Fig. 3(c). The scanning step used along the *z*-direction is 0.5 mm. The simulation results are also presented in Fig. 3(d). Excellent agreement between the experimental results and the corresponding theoretical expectations can be found. The figure shows that the THz wave was focused well on the preset position, which is located 3.84 mm away from the lens. Additionally, Fig. 3(e) shows the focal point profiles that correspond to the intensity distributions along the white dashed lines shown in Figs. 3(c) and 3(d), which both show good sinc shapes with a full width at half maximum of 352 μm. For comparison, the theoretical expectation was 313 μm, and the difference was likely to be caused by fabrication errors. To demonstrate the imaging performance of this metalens based on the CPC structure, three letter patterns (C, N, and U) that had been milled on a stainless steel sheet, as shown in Fig. 4a, were used as imaging objects. All THz images of the three letter patterns were obtained and are displayed clearly in Fig. 4(b–d), indicating that the designed metalens performs correctly.

**Figure 5:**
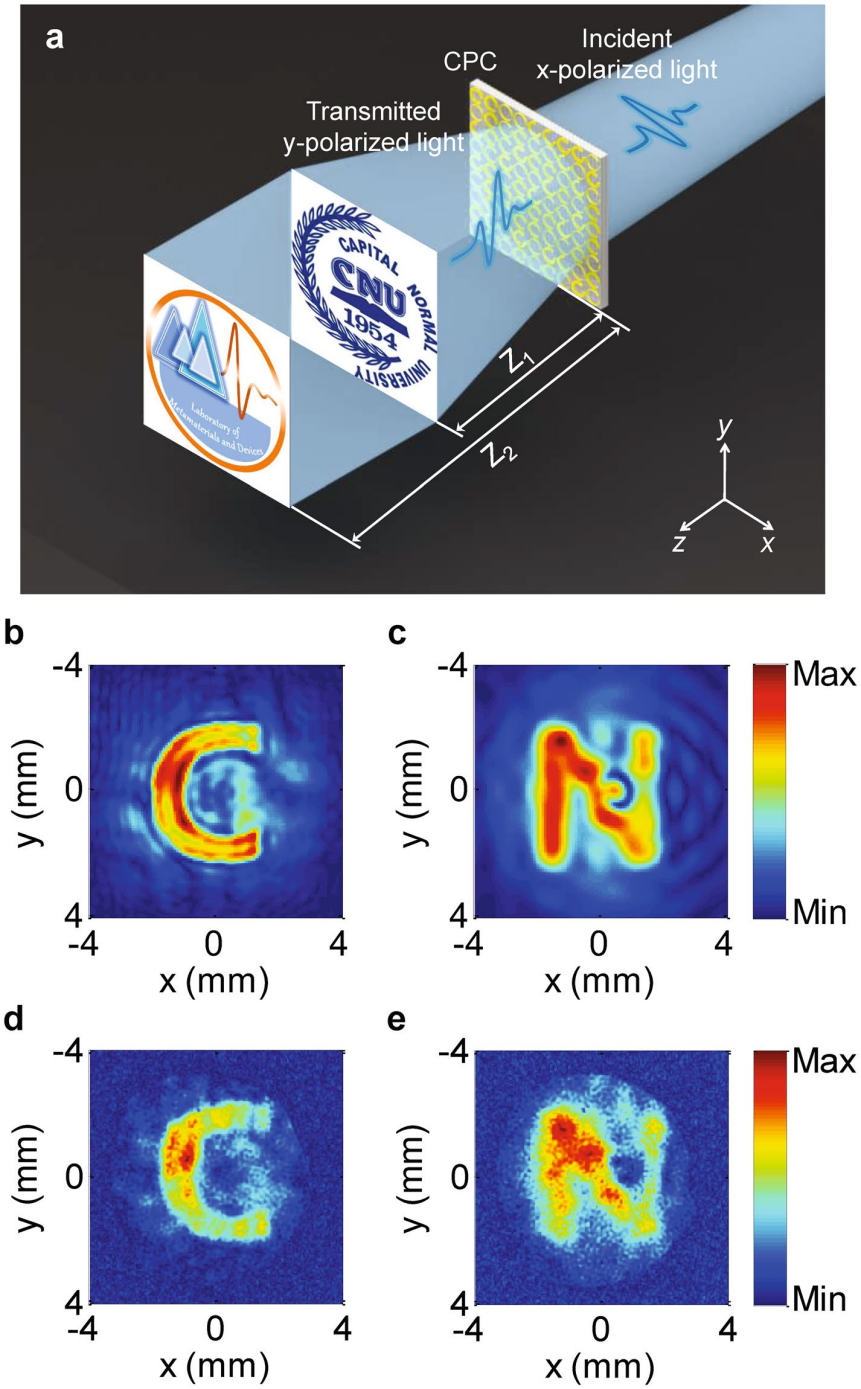
(**a**) Schematic of the hologram system. (**b**,**c**) Images intended to appear on the planes that are located 5 mm and 15 mm away from the structures, respectively. (**d**,**e**) Images generated by the hologram structures at the preset positions.

The CPC structure can also be used to realize phase holograms for arbitrary intensity distribution generation along the propagation direction. A schematic of such a system is shown in Fig. 5(a). When the device is illuminated using a 0.75 THz x-polarized wave, the transmitted y-polarized wave will then generate two predesigned hologram images at two different image planes; in this design, the patterns of letters C and N were designed to be generated at planes located 5 mm and 15 mm away from the sample, respectively. The CPC structure-based hologram device was designed using the simulated annealing algorithm and contains 128 × 128 cells, corresponding to a geometric area of 1.024 × 1.024 cm^2^. The hologram patterns that were recorded at 5 mm and 15 mm are shown in Fig. 5(d,e), respectively. The simulated results that were obtained using the Rayleigh-Somerfield diffraction integral are shown in Fig. 5(b,c), and indicate that the rebuilt patterns have slight cross-talk in each of the image planes. The measured “C” and “N” patterns agree well with the desired patterns in terms of their character shapes, sizes and locations at the different planes, thus demonstrating that the hologram provides good phase modulation.

## Conclusions

In conclusion, a high-efficiency CPC structure has been proposed and its physical mechanisms have been analyzed in detail. The fabrication procedure for this new type of CPC is relatively simple. Two high-efficiency THz devices based on the proposed bilayer metasurface CPC have been demonstrated experimentally. The performance levels of the designed devices have been measured carefully and indicate that the fabricated devices can perform their preset functions well. The high-efficiency device performance results indicate that the CPC structure can be applied to THz photonic devices, while the thicknesses of these devices make them highly suitable for system integration and miniaturization. This new approach may pave the way towards practical applications of THz metasurface devices.

## Methods

### Measurement method

In the experiment, a THz holographic imaging system^37^ is used to characterize the performance of the designed structure. The light source is a 100 fs ultrashort laser pulse with an 8-mm spot diameter, an 800-nm central wavelength and a 1-kHz repetition rate provided by a Ti: sapphire regenerative amplifier. The laser beam, which has an average power of 900 mW, is divided into two parts, which are the pump (880 mW) and probe (20 mW) beams used for generation and detection of the THz waves, respectively. The pump beam is used to impinge a <110> ZnTe crystal to generate a terahertz beam based on optical rectification. A horizontal polarized (x- polarized) THz beam with a diameter of 24 mm passing a THz polarizer for polarization-maintaining, and then impinges on the structure, and the scattered vertical polarized (y-polarized) THz beam is detected by another <110> ZnTe crystal. In the probe optical path, a half-wave plate (HWP) and a polarizer are used to modulate the probe beam polarization. Then, the probe beam with the y-polarization is reflected by a non-polarization beam splitter (BS) (T:R = 5:5) towards the sensor crystal. In the sensor crystal, the probe beam polarization is modulated by the THz field based on the Pockels effect, and the reflected probe beam is then captured by the imaging unit. The imaging unit consists of a 4 f system (two convex lenses), a quarter-wave plate (QWP), a Wollaston prism (WP), and a charge-coupled device (CCD) camera. The WP is used to split the probe beam into two beams with orthogonal polarizations, and the two images of the sensor crystal are then projected onto the CCD camera by the 4 f system. The THz complex field can be extracted using the balanced electro-optic detection technique. By varying the optical path difference between the THz beam and the probe beam, 25 temporal images are captured at each time delay, and the time window is 17 ps. The amplitude and phase information at the different frequencies can be extracted by performing Fourier transformations on the temporal signals at each pixel.
